# SERPINB10 contributes to asthma by inhibiting the apoptosis of allergenic Th2 cells

**DOI:** 10.1186/s12931-021-01757-1

**Published:** 2021-06-14

**Authors:** Yuqing Mo, Ling Ye, Hui Cai, Guiping Zhu, Jian Wang, Mengchan Zhu, Xixi Song, Chengyu Yang, Meiling Jin

**Affiliations:** Department of Pulmonary and Critical Care Medicine, Zhongshan Hospital, Fudan University, No. 180 Fenglin Road, Xuhui District, Shanghai, 200032 China

**Keywords:** Asthma, SERPINB10, Th2 response, Apoptosis

## Abstract

**Background:**

Serine peptidase inhibitor, clade B, member 10 (SERPINB10) contributes to allergic inflammation in asthma. However, its role in the T-helper type 2 (Th2) response of allergic asthma is not known. The goal of this study was to unveil the function of SERPINB10 in the Th2 response of allergic asthma and the mechanism by which SERPINB10 affects the viability of Th2 cells.

**Methods:**

Th2 cytokines and serum levels of house dust mite (HDM)-specific IgE in bronchoalveolar lavage fluid were examined by ELISA in an HDM-induced asthma model. The number and apoptosis of Th1 and Th2 cells in mouse lungs were measured by flow cytometry. Naïve CD4 T cells from patients with asthma were cultured under appropriate polarizing conditions to generate Th1 and Th2 cells. SERPINB10 expression in polarized Th1 and Th2 cells was quantified by real-time reverse transcription-quantitative polymerase chain reaction. SERPINB10 expression was knocked down in human CD4 T cells with lentivirus.

**Results:**

Knockdown of SERPINB10 expression significantly diminished HDM-induced Th2 cytokine secretion and level of HDM-specific IgE. After HDM exposure, SERPINB10-knockdown mice had diminished numbers of Th2 cells, but similar numbers of Th1 cells, compared with those in negative-control mice. Th2 cells of SERPINB10-knockdown mice were more susceptible to apoptosis than that of control mice. Stimulating T-cell receptors (TCRs) with anti-CD3 antibody caused upregulation of SERPINB10 expression in polarized Th2 cells, but not polarized Th1 cells. Knockdown of SERPINB10 expression resulted in fewer numbers and greater apoptosis of polarized Th2 cells.

**Conclusion:**

Our results suggest that SERPINB10 may contribute to allergic inflammation and the Th2 response of asthma by inhibiting the apoptosis of Th2 cells.

**Supplementary Information:**

The online version contains supplementary material available at 10.1186/s12931-021-01757-1.

## Background

Asthma is a common chronic inflammatory disease characterized by airway inflammation, airway hyperresponsiveness, mucus hyperproduction, and airway remodeling [1]. The Th2 cells and the cytokines that typify the Th2 cell subset underlies the inappropriate immune responses that characterize allergic asthma [2]. The “signature” cytokines of Th2 cells, interleukin (IL)-4, IL-5 and IL-13, have a central role in infiltration of eosinophils and increased secretion of mucus [3, 4].

Serine protease inhibitors (SERPINS) are a superfamily of homologous proteins that have important roles in inflammation, immune-system function, apoptosis regulation [5, 6], and metastasis of cancer cells [7]. Human SERPINS are divided into nine groups (A–I) called “clades” according to their sequence similarity. Expression of several members in clade B, such as SERPINB2, SERPINB3 and SERPINB4, is upregulated in allergic diseases, and they modulate Th1/Th2 responses [8–10]. Mice with SERPINB2 deficiency have a defective response from Th2 cytokines after nematode infection [8]. SERPINB3 and SERPINB4 in patients suffering from allergic disease control the viability of Th2 cells by exerting anti-apoptotic effects [11].

SERPINB10 is another member of clade B. Previously, we reported that SERPINB10 expression was increased in the bronchial epithelial cells of patients with asthma and was associated with airway eosinophilic inflammation. Knockdown of SERPINB10 expression can reduce airway hyperresponsiveness and airway eosinophilic inflammation in a murine model of asthma [12]. The role of SERPINB10 in the Th2 response of allergic asthma is not known. Schleef and colleagues reported that SERPINB10 can inhibit tumor necrosis factor α (TNF-α)-induced cell death [13]. Therefore, we hypothesized that SERPINB10 may participate in the Th2 response of allergic asthma by affecting the apoptosis of Th2 cells.

We investigated the effect of knockdown of SERPINB10 expression on the Th2 response and apoptosis of Th cells in a house dust mite (HDM)-induced model of asthma. We measured expression of SERPINB10 in polarized Th1 and Th2 cells derived from patients with asthma. We assessed the effect of SERPINB10 on the apoptosis of Th cells in vitro.

## Methods

### Mouse model

Female C57BL/6 J mice (6–8 weeks) were purchased from JSJ Laboratory (Shanghai, China) and bred under specific pathogen-free conditions at the animal center of Zhongshan Hospital, Fudan University (Shanghai, China). All experimental protocols were approved by the Animal Care and Use Committee of Zhongshan Hospital. Mice were anesthetized with isoflurane and administered, via the intratracheal route, adeno-associated virus (AAV) (30 µL; 6.32 × 10^12^ viral particles/mL; Vigene Biosciences, Shandong, China) containing SERPINB10 short hairpin (sh)RNA or scrambled shRNA. The sequence of SERPINB10 shRNA was GCAGAACCACAATCTGTTAACTTCAAGAGAGTTAACAGATTGTGGTTCTGCTTTTTT. After 2 weeks, the mice were sacrificed to evaluate the knockdown efficiency of Serpinb10 AAV. Another batch of mice was used to establish an HDM asthma model. Mice were randomly divided into three groups: control group, asthma group and knockdown asthma group. Mice were sensitized by intranasal instillation of HDM extract (10 μg; Greer Laboratories, Lenoir, NC, USA) in 40 μL of phosphate-buffered saline (PBS) on days 0, 1, and 2. From day-8 to day-12, mice were challenged daily by intranasal administration of HDM (10 μg in 40 μL of PBS). Control mice were given, via the intranasal route, 40 μL of PBS during sensitization and challenge phases. Two weeks before the first sensitization, mice were administered 30 µL AAV containing SERPINB10 shRNA or scrambled shRNA. Mice were sacrificed for evaluation on day-14. There were five to six mice per group for each independent experiment.

### RT-qPCR and Western blotting

RT-qPCR and Western blotting were performed as our previous study [12] and the protocol is given in the Additional file 2: Materials and Methods. The primer sequences of all genes for PCR are listed in Additional file 1: Table S1.

### Analyses of bronchoalveolar lavage fluid (BALF)

BALF was collected according to a method described previously [14]. Briefly, the trachea was cannulated through a 22-inch intravenous catheter and the lungs were lavaged with a total volume of 1 mL PBS for three successive aspirations to obtain BALF. The BALF was centrifuged at 500×*g* for 8 min at 4 ℃. The cell-free supernatant was collected for cytokine analyses using ELISA kits. Cell pellets were resuspended in PBS and the total cell number was counted using CellDrop® (DeNovix, Wilmington, DE, USA). Cells were analyzed by flow cytometry using PE-conjugated anti-SiglecF (eBioscience, San Diego, CA, USA), FITC-conjugated anti-CD3 (BD Biosciences, San Jose, CA, USA), APC-conjugated anti-CD11c (Multiscience, Zhejiang, China), FITC-conjugated anti-CD19 (BioLegend, San Diego, CA, USA), Percp-cy5.5-conjugated anti-Ly6G (BioLegend) and PE-cy7-conjugated anti-MHC II (BioLegend). The flow cytometry gating strategy was according to a method described previously [15].

### Flow cytometry of lung tissues

We wished to calculate the number of Th cells in lungs. Lung tissues were immersed in Hank’s medium containing collagenase IV (1 mg/mL) and DNase I (20 μg/mL). Lung tissues were ground using a gentleMACS® Dissociator (Miltenyi Biotec, Bergisch Gladbach, Germany) and then incubated with shaking at 100 rpm for 30 min at 37 ℃. Digested tissues were filtered through 70-μm nylon mesh, treated with red blood cell lysis buffer, and washed with staining buffer (PBS containing 2% fetal bovine serum). Cells were first incubated with purified anti-mouse CD16/32 (eBioscience) for 10 min (to block Fc receptors) and then stained with a mixture of Percp-cy5.5-conjugated anti-CD3e (BioLegend), BV510-conjugated anti-CD4 (BD Bioscience) and APC-cy7-conjugated anti-CD45 (BioLegend) for 30 min on ice. For intracellular staining, cells were fixed and permeabilized with Transcription Factor Buffer Set (BD Pharmingen, Franklin Lakes, NJ, USA) before addition of AF647-conjugated anti-GATA3 (BioLegend), BV421-conjugated anti- T-bet (BD Pharmingen) and PE-conjugated anti-active caspase-3 (BD Pharmingen). After washing, samples were analyzed by an Arial III flow cytometer (BD Biosciences) and data were analyzed using FlowJo® (Tree Star, Ashland, OR, USA).

### Histology

The left lobes of mouse lungs were perfused with 0.3 ml 4% paraformaldehyde and then the left lobes of mouse lungs were isolated and fixed in 4% paraformaldehyde. Paraffin-embedded 5-μm lung sections were stained with hematoxylin and eosin (H&E) and periodic acid Schiff to assess infiltration of inflammatory cells, goblet-cell metaplasia, and mucus production.

### Measurement of levels of HDM-specific IgE and cytokines

Mouse ELISA kits for IL-4, IL-5 and IL-13 in BALF (R&D Systems, Minneapolis, MN, USA) and HDM-specific IgE in serum (JingKang Biotech, Shanghai, China) were used to measure protein expression according to manufacturer instructions. The cytokines in supernatants produced by polarized T cells were stained using the LEGENDplex® panel for human Th cytokines (BioLegend) and measured by flow cytometry.

### Clinical samples

Patients with asthma were diagnosed by a physician according to Global Initiative for Asthma guidelines. All asthma patients were clinically diagnosed with symptomatic asthma and demonstrated evidence of a hyperresponsive airway (provocative dose of methacholine causing a 20% drop in FEV1 < 2.5 mg) and/or bronchodilator responsiveness (> 12% improvement in FEV1% predicted following inhalation of 200 μg of salbutamol). None of the patients had ever received inhaled or oral corticosteroid or leukotriene antagonist. Demographic information (Table 1) and blood samples from 16 patients were collected for study. Written informed consent was obtained from all patients. This study was approved by the ethics committee of Zhongshan hospital, Fudan University.

**Table 1:** Subject characteristics

	Asthma (n = 16)
Age, year	35 (18,66)
Sex, M:F	6:10
Body mass index	23.8 ± 3.6
FEV_1_, % predict	90.6 ± 13.6
FENO, ppb	40.5 (16.25, 88.25)
Total IgE, IU/ml	254.0 (231.3, 340.5)

Values were presented as mean (range) or mean ± SD or median with interquartile range. FEV_1_, forced expiratory volume in the first second; FENO, fraction of exhaled nitric oxide

### Polarization of Th1 and Th2 cells in vitro

Heparinized venous blood was collected from patients with asthma. Then, it was diluted (1:1) with PBS and layered on Lymphoprep® (StemCell Technologies, Vancouver, Canada) density-gradient medium and centrifuged for 20 min at 800×*g* at room temperature. The layer of peripheral-blood mononuclear cells was collected, washed and resuspended in RoboSep® Buffer (StemCell Technologies). Naïve CD4^+^CD45RA^+^CD45RO^−^ T cells were negatively selected and enriched using EasySep® Human Naïve CD4^+^ T Cell Isolation Kit II (StemCell Technologies) according to manufacturer instructions. The purity of the final isolated fraction (as determined by flow cytometry using FITC-conjugated anti-CD4, PE conjugated anti-CD45RA and APC-conjugated anti-CD45RO (BioLegend)) was 97%. Purified naïve CD4^+^ T cells were cultured in ImmunoCult®-XF Cell Expansion Medium (StemCell Technologies) and stimulated with plate-bound anti-CD3 (2 μg/mL) and anti-CD28 (4 μg/mL; Peprotech-BioGems, Westlake Village, CA, USA). ImmunoCult Human Th1 Differentiation Supplement (StemCell Technologies) and ImmunoCult Human Th2 Differentiation Supplement (StemCell Technologies) were added to direct the differentiation of Th1 and Th2 cells, respectively. After 3–4 days, cells were expanded under identical conditions in the absence of anti-CD3 and anti-CD28. Then, cells were re-stimulated every 7 days. If required, cells were activated with Leukocyte Activation Cocktail (BD Pharmingen) for 6 h.

### Knockdown of SERPINB10 expression in T cells

Lentivirus containing SERPINB10 shRNA or scrambled shRNA were used to transduce CD4^+^ T cells. The sequence of SERPINB10 shRNA was GCCTGTTAACTTTGTGGAA. Naïve CD4^+^CD45RA^+^CD45RO^−^ T cells were stimulated with plate-bound anti-CD3 and anti-CD28. After 48 h, they were transduced with lentivirus (multiplicity of infection = 100) by centrifugation at 500×*g* for 90 min at room temperature in polybrene (6 μg/mL) and then cultured at 37℃ in a chamber containing 5% CO_2_. After 3 days, cells were analyzed by flow cytometry for expression of CD4 and green fluorescent protein (GFP). If required, GFP^+^ cells were sorted by flow cytometry and cultured under polarization conditions.

### Statistical analyses

Data are the mean ± SEM and were analyzed using Prism 8 (Graph Pad, San Diego, CA, USA). Differences were assessed using the unpaired Student’s *t*-test between two groups, and one-way analysis of variance with Tukey’s multiple comparison test among three groups. *P* < 0.05 was considered significant.

## Results

### Knockdown of SERPINB10 expression alleviates HDM-induced airway inflammation and the Th2 response in a murine model of asthma

**Fig. 1:**
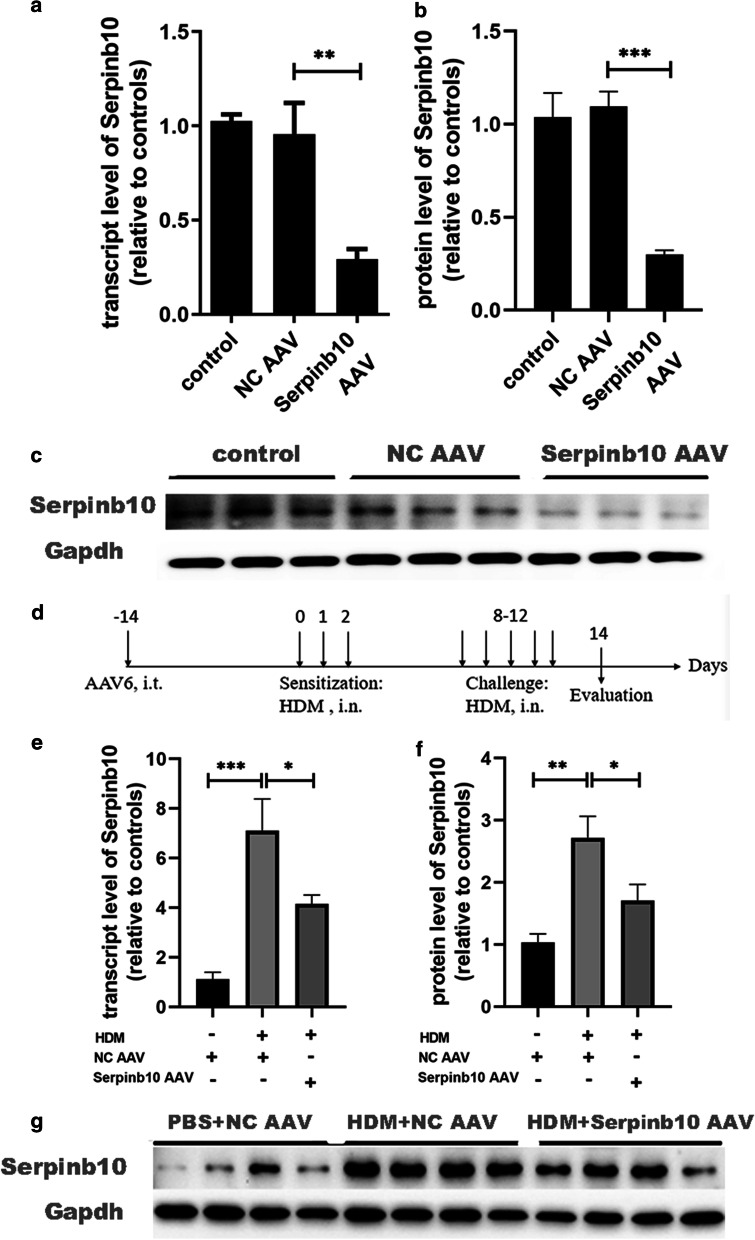
Adeno-associated virus against Serpinb10 can inhibit the expression of baseline and HDM-induced Serpinb10 in mouse lung tissues. **a** Serpinb10 transcript levels in mouse lungs after 2 weeks of AAV treatment were determined by quantitative PCR. **b** and **c** Serpinb10 protein levels in mouse lungs after 2 weeks of AAV treatment were determined by western blotting. **d** C57BL/6 J mice were sensitized with 10 μg HDM on days 0,1,2 and challenged with 10 μg HDM daily from days 8–12. Mice were euthanized on day 14 for analysis. Control mice received PBS i.n. for sensitization and challenge. Serpinb10 AAV or negative control AAV was administered to the airway of mice 2 weeks before sensitization. **e** Serpinb10 transcript levels in mouse lungs on day 14 were determined by quantitative PCR. **f** and **g** Serpinb10 protein levels in mouse lungs on day 14 were determined by western blotting. Data were combined from three experiments (n = 12–14). One-way ANOVA was used. *, *P* < 0.05; **, *P* < 0.01; ***, *P* < 0.001

We wished to investigate the role of SERPINB10 in the Th2 response of allergic asthma. We used an HDM-induced asthma model and knocked down SERPINB10 expression by transfection with AAV against SERPINB10 (Fig. 1a). On day-0, some mice were euthanized and lung tissues were collected for analyses. Expression of protein and transcript levels of SERPINB10 were suppressed by 74.2% by SERPINB10 AAV. Negative control AAV had no effect on *SERPINB10* expression (Fig. 1b, c). On day-14, expression of protein and transcript levels of SERPINB10 were increased significantly after HDM sensitization and challenge, and were suppressed after knockdown of SERPINB10 expression (Fig. 1d, e). Our results suggested that SERPINB10 AAV could inhibit baseline and HDM-induced SERPINB10 expression in mouse lungs effectively and continuously.

**Fig. 2:**
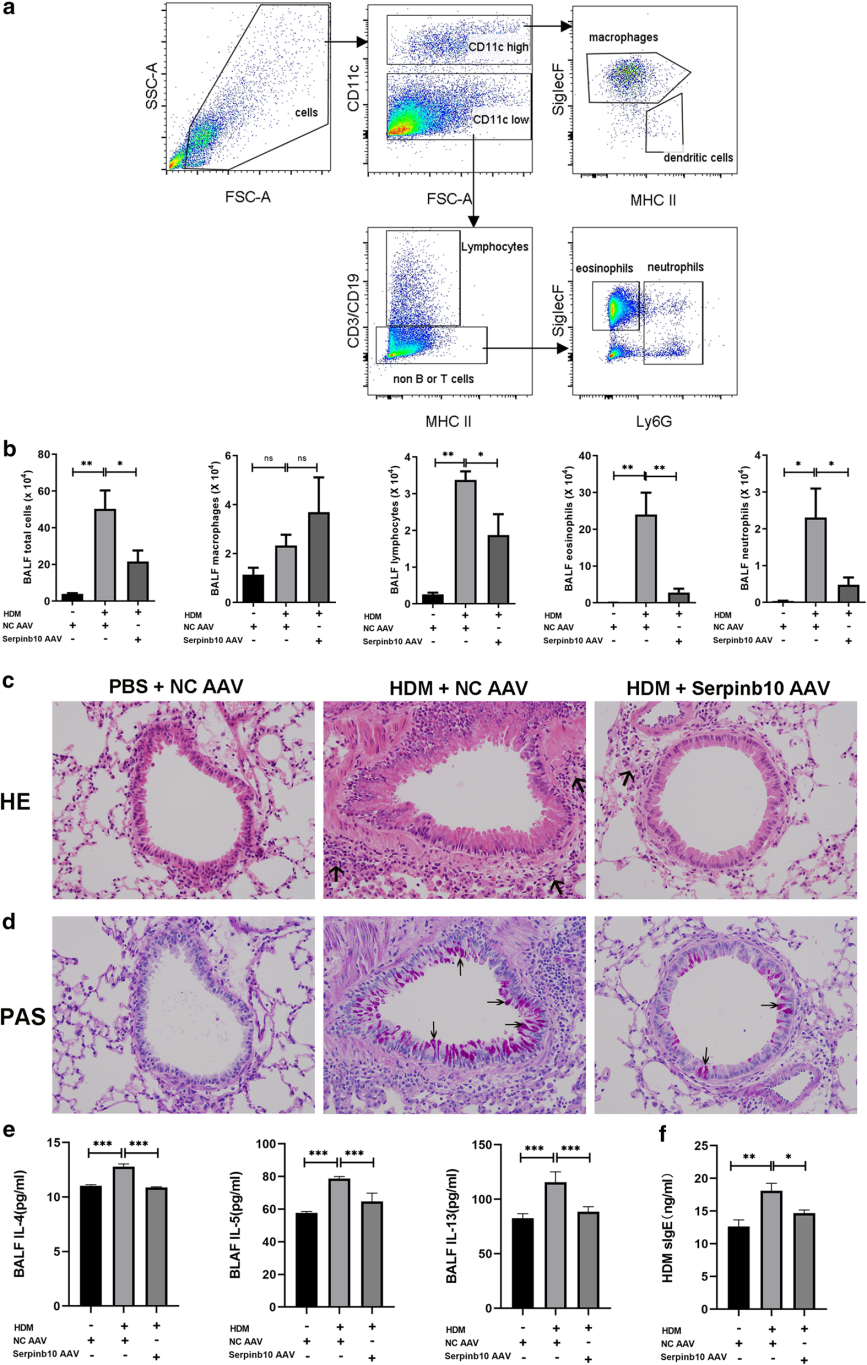
Knockdown of Serpinb10 expression reduced HDM-induced allergic asthma. **a** flow cytometry gating strategy for inflammatory populations in bronchoalveolar lavage fluid (BALF). Live cells can be separated in a CD11c^high^ and a CD11c^low^ cell population. Within the CD11c^high^ cell population, gate for macrophages (SiglecF^high^ and MHCII^low^) and dendritic cells (SiglecF^low^ and MHCII^high^). Within the CD11c^low^ cell population, gate for lymphocytes and non-lymphocytes based on CD3/CD19 and MHCII expression. Within the CD11c^low^ CD3/CD19^neg^ cell population, gate for neutrophils (Ly-6G^high^) and eosinophils (Ly-6G^low^, SiglecF^high^). **b** counts for total cells, macrophages, lymphocytes, eosinophils and neutrophils in BALF. **c** lung sections were stained with hematoxylin–eosin. The arrows indicate the peri-bronchial infiltration of inflammatory cells; original magnification, × 400. **d** periodic acid-Schiff (PAS) staining of representative lung sections, arrows indicate PAS positive cells; original magnification, × 400. **e** the protein levels of IL-4, IL-5 and IL-13 in BALF were determined by ELISA. **f** HDM-specific IgE was measured in mouse sera using ELISA. Data in (**b**, **e**, **f**) were pooled from three independent experiments. n = 12–14. *, *P* < 0.05; **, *P* < 0.01; ***, *P* < 0.001

We used flow cytometry to analyze the inflammatory cells in BALF. The gating strategy is shown in Fig. 2a. The number of total cells, lymphocytes, eosinophils, and neutrophils was increased significantly after exposure to HDM, thereby suggesting that HDM induced allergic inflammation in mouse airways. Knockdown of SERPINB10 expression reduced the number of total cells, lymphocytes, eosinophils, and neutrophils in BALF (Fig. 2b). In support of these observations, histology of lung tissues showed that mice with knockdown of SERPINB10 expression had less peri-bronchial infiltration of inflammatory cells after HDM exposure (Fig. 2c). Goblet-cell hyperplasia and mucus secretion were increased after HDM challenge but were reduced significantly after knockdown of SERPINB10 expression (Fig. 2d). Expression of the Th2 cytokines IL-4, IL-5 and IL-13 in BALF was increased in mice challenged with HDM compared with those challenged with PBS. Knockdown of SERPINB10 expression diminished HDM-induced secretion of Th2 cytokines significantly (Fig. 2e). We also documented reduced expression of HDM-specific IgE in the sera of SERPINB10-knockdown mice (Fig. 2f). Our data suggested that SERPINB10 contributed to HDM-induced airway inflammation and the Th2 response.

### The number of Th2 cells is diminished after challenge in SERPINB10-knockdown mice

**Fig. 3:**
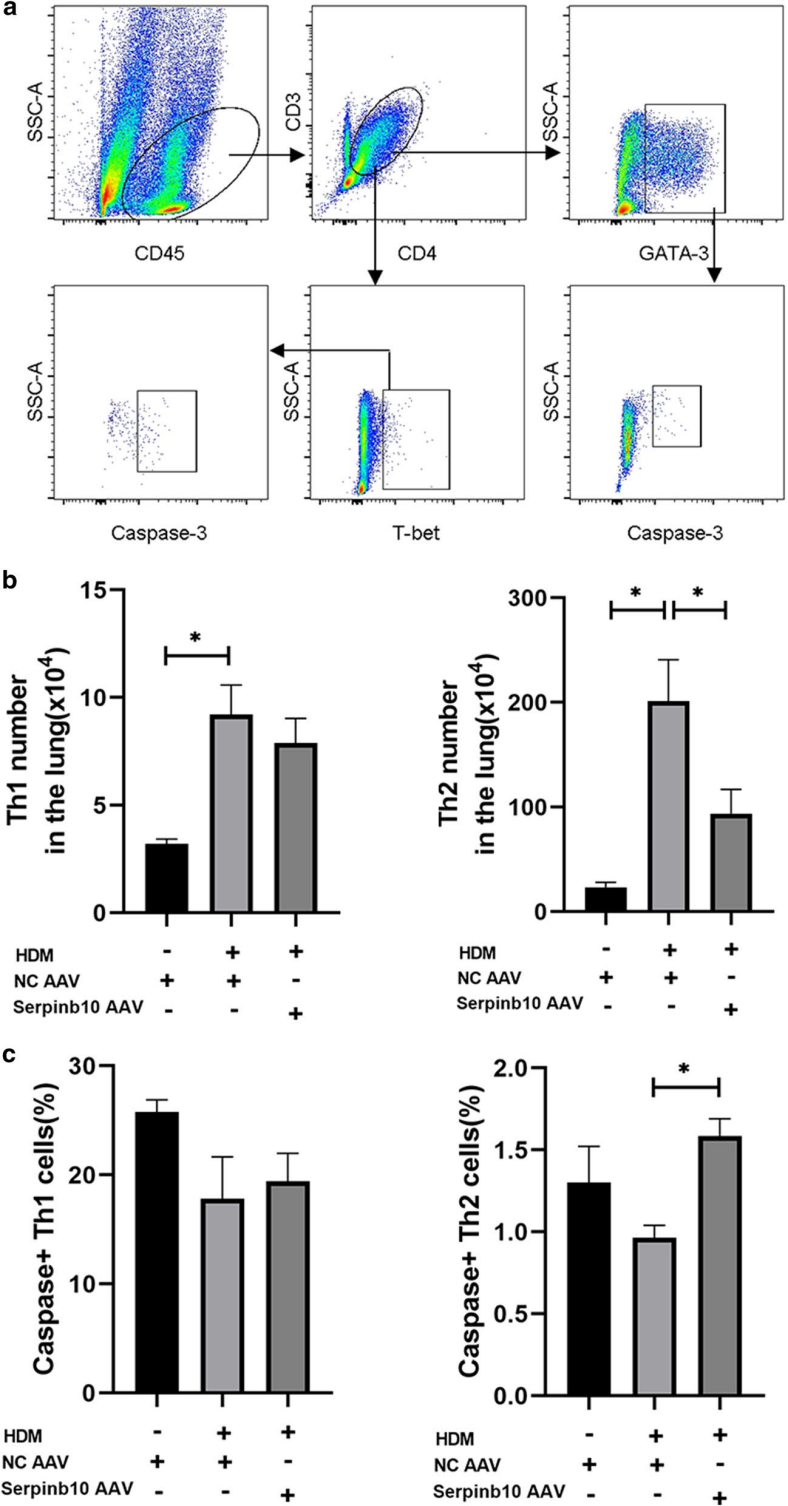
Serpinb10-knockdown mice had diminished numbers of Th2 cells after challenge. **a** Gating strategy to identify Th cells and their apoptosis in mouse lungs. Live cells can be separated in a CD45^+^ and a CD45^−^ cell population. Within the CD45^+^ cell population, gate for CD4 T cells (CD3^+^ CD4^+^). Within the CD4 T cells, gate for Th1 cells (T-bet^+^) and Th2 cells (GATA-3^+^). Within the Th1 cells and Th2 cells, gate for apoptosis cells (Active Caspase-3^+^) respectively. **b** The numbers of Th1 and Th2 cells in mouse lungs. **c** percentage of caspase-3-positive Th1 and Th2 cells in mouse lungs. Data were combined from three experiments. n = 12–14. *, *P* < 0.05.

We further explored the reasons for the defective Th2 response in SERPINB10-knockdown mice. Lung tissues were prepared into a single-cell suspension and the number of Th cells in lung tissue measured by flow cytometry (Fig. 3a). The number of Th1 and Th2 cells in lung tissues was increased in mice challenged with HDM compared with those challenged with PBS. However, the increase in the number of Th2 cells was much higher than that of Th1 cells. After HDM exposure, SERPINB10-knockdown mice had diminished numbers of Th2 cells, but similar numbers of Th1 cells, compared with those of negative-control mice (Fig. 3b). Caspase-3 is known as an executioner caspase in apoptosis because of its role in coordinating the destruction of cellular structures such as DNA fragmentation or degradation of cytoskeletal proteins.Active caspase-3 is a marker for cells undergoing apoptosis [16]. Intracellular staining for activated caspase-3 revealed that more Th1 cells underwent apoptosis compared with Th2 cells. However, a significant difference in the percentage of activated caspase-3-positive Th1 cells was not observed between SERPINB10-knockdown mice and control mice after HDM exposure. In contrast, Th2 cells in SERPINB10-knockdown mice were more susceptible to apoptosis than those in control mice (Fig. 3c). Our data suggested that SERPINB10 may contribute to airway inflammation and the Th2 response of asthma by regulating apoptosis of Th2 cells.

### SERPINB10 is required for development of Th2 cells

**Fig. 4:**
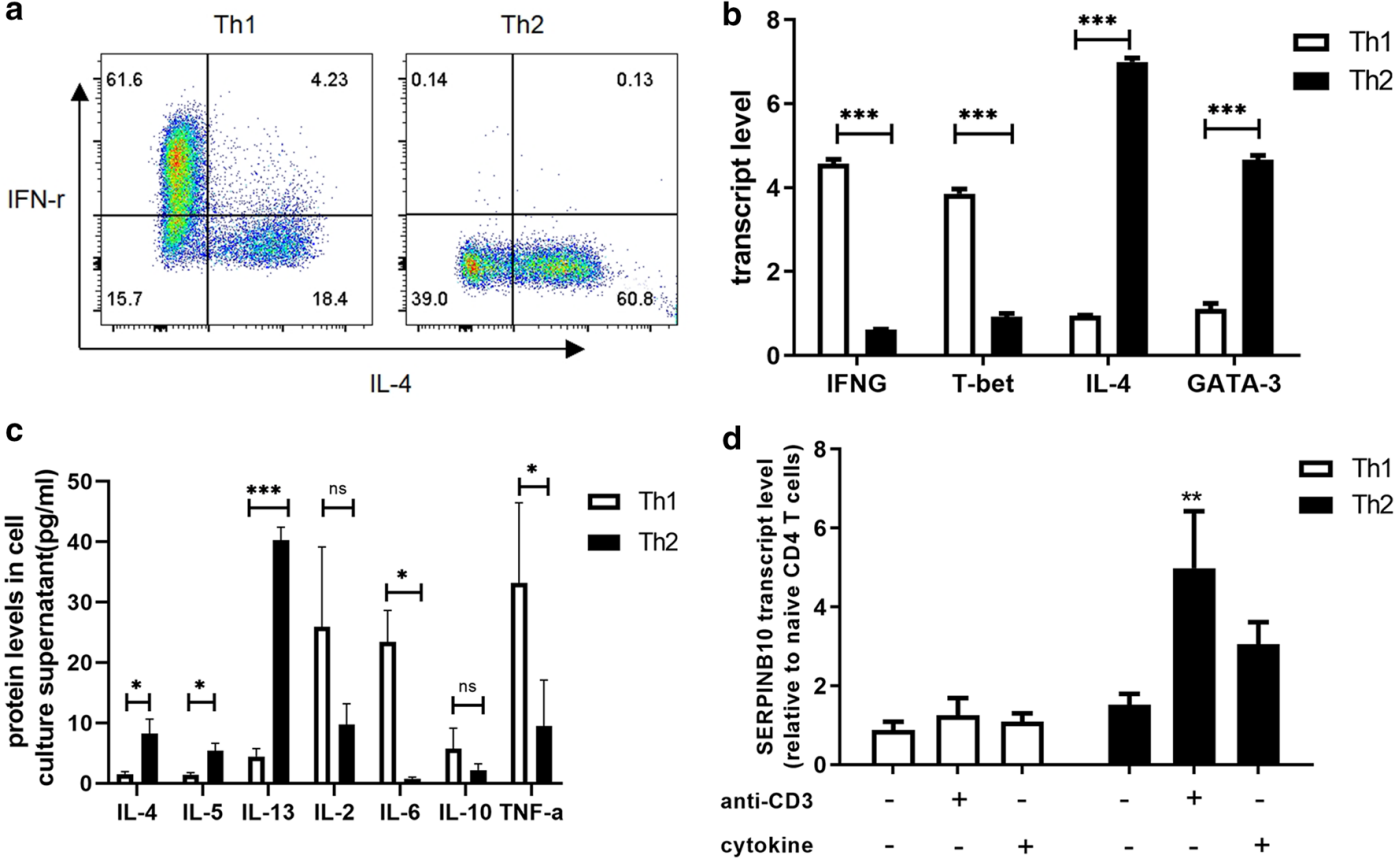
Expression level of SERPINB10 in human Th cells. **a** flow cytometric plots for Th1 or Th2 polarized CD4 T cells from patients with asthma after intracellular staining for IFN-γ or IL-4. **b** the transcript level of IFNG, T-bet, IL-4 and GATA-3 in polarized Th1 and Th2 cells was measured using quantitative PCR. **c** cytokine levels in culture supernatant of polarized Th1 or Th2 cells were measured by LEGENDplex human Th cytokine panel. **d** SERPINB10 transcript level (fold change over naïve CD4 T cells) in Th1 or Th2 cells after no stimulation or stimulation with anti-CD3 or cytokine (IFN-γ for Th1 and IL-4 for Th2). n = 16 patient samples. Data were means ± SE. *, *P* < 0.05; **, *P* < 0.01; ***, *P* < 0.001; ns, not significant

We further investigated the role of SERPINB10 in polarized Th2 cells in vitro. Naïve CD4 T cells (CD4^+^CD45RA^+^CD45RO^−^) were magnetic beads sorted from the peripheral blood of asthma patients and then cultured under appropriate polarizing conditions to generate Th1 and Th2 cells. Upon completion of polarization, cells were activated with Leukocyte Activation Cocktail and verified using intracellular staining and flow cytometry to detect signature cytokines (Fig. 4a).

Approximately 60% of CD4 T cells could be polarized into Th1 or Th2 cells under appropriate polarization conditions (Fig. 4a). Polarized Th1 cells were confirmed by upregulation of transcript levels of interferon (IFN)-γ and T-bet compared with that in polarized Th2 cells. In contrast, polarized Th2 cells showed upregulated transcript levels of IL-4 and GATA-3 compared with those in polarized Th1 cells (Fig. 4b). Cytokine levels in cell-culture supernatants were measured by LEGENDplex. Polarized Th1 cells secreted more IL-6 and TNF-α than polarized Th2 cells. IL-2 and IL-10 had similar expression trends but were not significant. Polarized Th2 cells produced more IL-4, IL-5 and IL-13 than polarized Th1 cells (Fig. 4c).

Next, we determined whether the SERPINB10 transcript level was increased in Th1 or Th2 cells. Stimulation of T-cell receptors (TCRs) with anti-CD3 antibody caused upregulation of SERPINB10 expression in polarized Th2 cells but not in polarized Th1 cells. Moreover, stimulation through the IFN-γ receptor did not induce an increase in SERPINB10 transcript levels in polarized Th1 cells, whereas stimulation of IL-4 receptors resulted in upregulation of SERPINB10 expression in polarized Th2 cells, though not significantly (Fig. 4d). Our data demonstrated that stimulation through TCRs resulted in upregulation of SERPINB10 expression in polarized Th2 cells, but not in Th1 cells.

### Th2 cells with knockdown of SERPINB10 expression are susceptible to apoptosis

**Fig. 5:**
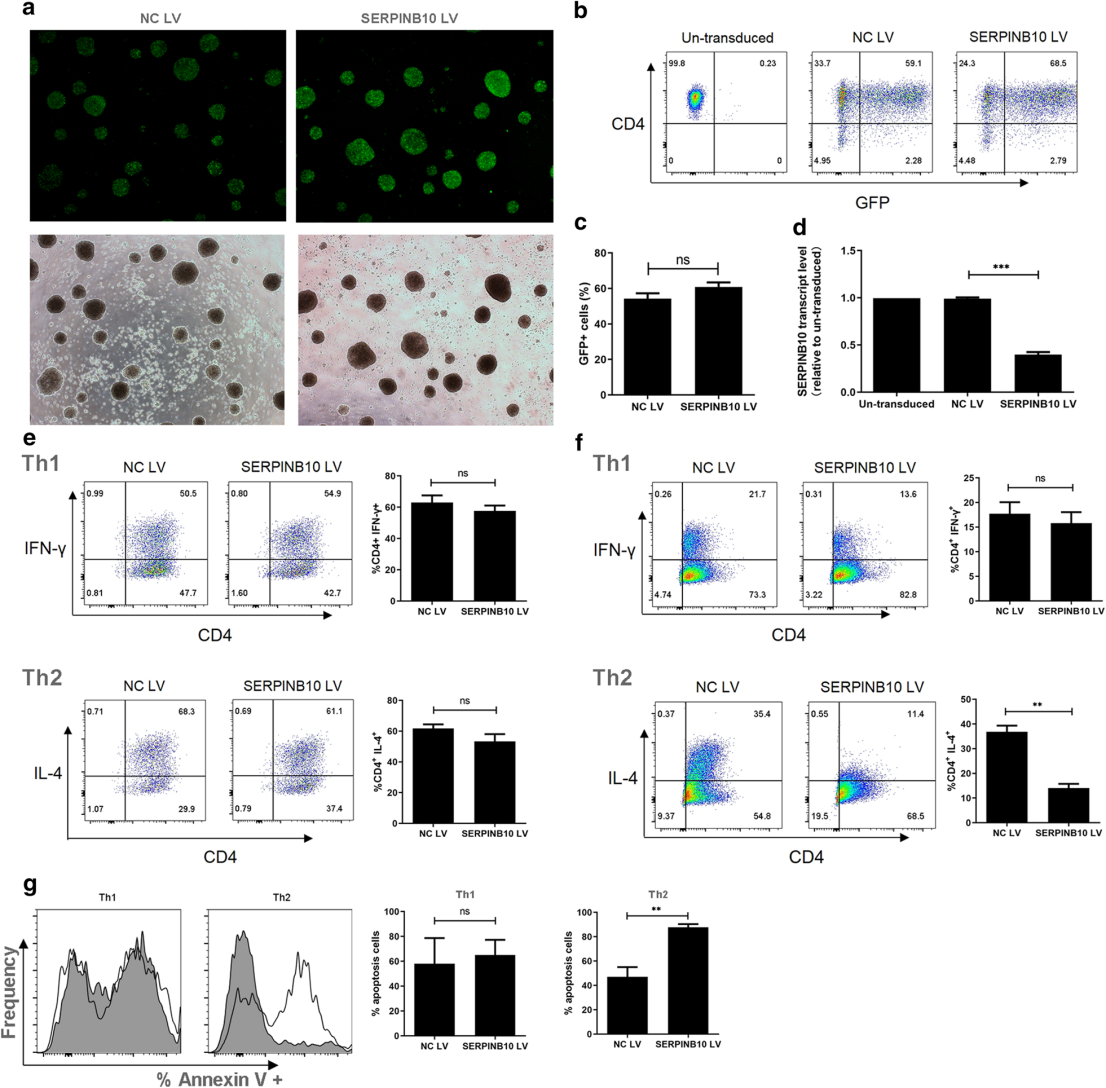
Knockdown of SERPINB10 expression affects survival of Th2 cells. **a** representative light micrograph of lentivirus (LV)-transduced CD4 T cells (top and bottom panels are pictures of the same cells in fluorescence mode and normal light mode respectively). **b** and **c** flow cytometric data for green fluorescent protein (GFP)^+^ percentage of lentivirus-transduced CD4 T cells. **d** SERPINB10 transcript level in lentivirus-transduced CD4 T cells (fold change compared with ACTB housekeeping gene). **e** percentage of IFN-γ^+^ or IL-4^+^ polarized CD4 cells upon completion of Th1 and Th2 polarization. **f** percentage of IFN-γ^+^ or IL-4^+^ polarized CD4 cells after completion of polarization and rested for another 8 days. **g** flow cytometric histogram for negative control (solid histogram) or SERPINB10-knockdown (line) Th1 and Th2 cells after staining with AnnexinV. Percentage of AnnexinV-positive cells were shown in histograms. Data were pooled from three independent experiment; n = 4 wells per group for each independent experiment. Data were mean ± SE. *, *P* < 0.05; **, *P* < 0.01; ***, *P* < 0.001; ns, not significant

To clarify further the functional importance of upregulation of SERPINB10 expression in Th2 cells, we sorted naïve CD4 T cells from the peripheral blood of asthma patients and transduced them with lentivirus encoding control shRNA or shRNA specific for SERPINB10. Transduced cells were identified based on GFP expression by microscope observation and flow cytometry (Fig. 5a, b). Approximately 60% of total cells were transduced with lentivirus (Fig. 5c). Levels of SERPINB10 transcripts decreased by 60% after transduction of lentivirus (Fig. 5d). GFP^+^ cells were sorted by flow cytometry and then cultured under polarization conditions. Upon completion of polarization, we did not observe a significant difference in the percentage of Th1 cells between the SERPINB10-knockdown group and control group. Similarly, there was no significant difference in the percentage of Th2 cells between these two groups (Fig. 5e). However, when cells were expanded under an identical culture condition and rested until day-8, the percentage of Th2 cells in the SERPINB10-knockdown group was decreased significantly compared with that in the control group (Fig. 5f). Phosphatidylserine expression on plasma membranes was measured by Annexin-V staining and revealed that Th2 cells with knockdown of SERPINB10 expression were more susceptible to apoptosis than control Th2 cells (Fig. 5g). However, a significant difference in the percentage of Th1 cells (Fig. 5f) and percentage of Annexin V-positive Th1 cells (Fig. 5g) between two groups was not observed. Our data demonstrated that SERPINB10 could protect Th2 cells from apoptosis.

## Discussion

We demonstrated that knockdown of SERPINB10 expression alleviated allergic inflammation and Th2 responses in an HDM model of asthma. SERPINB10-knockdown mice had diminished numbers of Th2 cells and greater apoptosis after HDM challenge. SERPINB10 was expressed in polarized Th2 cells from patients with asthma. SERPINB10 expression was upregulated in polarized Th2, but not Th1, cells after TCR stimulation. Knockdown of SERPINB10 expression in human polarized Th cells resulted in significant impairment of survival of Th2 cells. Our results indicate that SERPINB10 contributes to the survival of Th2 cells in mice and humans.

One genome-wide association study found that many *cis*-expression quantitative trait loci across SERPINB10 were in the chromatin-interaction regions of transcriptional/enhancer activity in some immune cells. Those findings indicate that SERPINB10 has central roles in the immune response [17]. A review by Ashton and colleagues showed that several SERPINS in clade B can control the recognition of antigens and effector functions of T lymphocytes by promoting their viability [18].

Here we showed, for the first time, that SERPINB10 has a protective effect upon Th2 cells in people with asthma and in mice challenged by HDM. The role of SERPINB10 in Th2 cells was significantly different from its role in other T cells. In the present study, there was no evidence that SERPINB10 had an effect on the survival of Th1 cells. However the use of a more physiological relavant model for in vivo Th1 development than HDM model would be useful to more fully explore this possibility. We speculate that the specific anti-apoptotic effect of SERPINB10 upon Th2 cells was due to upregulation of SERPINB10 expression through TCR stimulation, whereas SERPINB10 expression in Th1 cells was not upregulated after stimulation and, therefore, Th1 cells were not affected. However, the mechanism by which TCR signals upregulate SERPINB10 expression in Th2 cells, but not in Th1 cells, merits further study.

One in vitro polarization study demonstrated that Th2 cells are relatively resistant to activation-induced cell death compared with Th1 cells [19]. We found that, compared with Th2 cells, more Th1 cells suffered apoptosis. Our data clearly show that SERPINB10 protects Th2 cells, but not Th1 cells, from apoptosis. Our findings suggest that SERPINB10 may promote allergic asthma by inhibiting the apoptosis of Th2 cells.

The persistence of Th2 cells and increased apoptosis of Th1 cells contributes to atopic and allergic diseases [20–22]. Eliminating Th2 cells by inducing apoptosis may help to alleviate allergic asthma [23, 24]. We showed that targeting of SERPINB10 expression by shRNA significantly decreased the number of Th2 cells in mouse lungs, and promoted the apoptosis of Th2 cells in patients suffering from asthma. Krug and coworkers found that cleaved and inactive GATA-3 mRNA can attenuate the asthmatic response and Th2-regulated inflammatory response in patients with allergic asthma [25]. Taken together, these findings provide support for the concept of targeting Th2 cells by knockdown of SERPINB10 expression.

Our study had three main limitations. First, in vivo data were based on knockdown of SERPINB10 expression in mouse lungs, but not in SERPINB10-knockout mice. Second, we sorted naïve CD4 T cells from the peripheral blood of asthma patients and explore the expression and the role of SERPINB10 in Th1 and Th2 cells. The role of SERPINB10 in helper T cells of healthy subjects still needs to be further clarified. Third, Th2 cytokines such as IL-4, IL-5 and IL-13 can also be secreted by group-2 innate lymphoid (ILC2) cells, which are activated at an early stage of sensitization and promote innate allergic inflammation and the adaptive Th2 response [26, 27]. Further studies will clarify if SERPINB10 protects ILC2 cells from apoptosis in allergic asthma.

## Conclusions

We demonstrated, for the first time, that SERPINB10 protects Th2 cells from apoptosis. SERPINB10 may represent a therapeutic target for alleviation of the Th2 response in asthma.

## Supplementary Information

**Additional file 1: Table S1.** Primers for quantitative PCR.

**Additional file 2**. The protocol of RT-qPCR and Western blotting.

## Data Availability

All data generated or analysed during this study are included in this article and its Additional files 1, 2.

## Abbreviations

SERPINB10: Serine peptidase inhibitor, clade B, member 10
Th2: T-helper type 2
HDM: House dust mite
TCRs: T-cell receptors
BALF: Bronchoalveolar lavage fluid
RT-qPCR: Real-time reverse transcription-quantitative polymerase chain reaction
GATA3: GATA binding protein 3
T-bet: T-box expressed in T cells
FEV_1_: Forced expiratory volume in the first second
FENO: Fraction of exhaled nitric oxide
ELISA: Enzyme-linked immunosorbent assay
MHC II: Major histocompatibility complex II
