# Supplementary material for: SERPINB10 contributes to asthma by inhibiting the apoptosis of allergenic Th2 cells
Source: Respir Res. 2021 Jun 14;22:178. doi: 10.1186/s12931-021-01757-1 (PMC8201873; doi:10.1186/s12931-021-01757-1)
Supplement: Supplementary file 1 — Additional file 1: Table S1. Primers for quantitative PCR. [file 12931_2021_1757_MOESM1_ESM.docx]

Supporting information

Supplemental Table 1. Primers for quantitative PCR

| Gene | Sequence |
| --- | --- |
| Mouse gene |  |
| *Actb* |  |
| Forward | GTGACGTTGACATCCGTAAAGA |
| Reverse | GCCGGACTCATCGTACTCC |
| *Serpinb10* |  |
| Forward | TCTGCTGAGGGTAGAAACATCT |
| Reverse | GGGTCTGGAAGTCGGACTGTA |
| Human gene |  |
| *ACTB* |  |
| Forward | CATGTACGTTGCTATCCAGGC |
| Reverse | CTCCTTAATGTCACGCACGAT |
| *SERPINB10* |  |
| Forward | AGCCCAACGATGACTACTTACT |
| Reverse | ACCCAAGAGTTGATGTCCTTTCT |
| *IFNG* |  |
| Forward | CTG ACT TGA ATG TCC AAC GC |
| Reverse | CGA AAC AGC ATC TGA CTC CTT |
| *T-bet* |  |
| Forward | CCC ATC CTC TGC CCT AAC TA |
| Reverse | CTC CTT CAT GCC CAA GAC TT |
| *IL-4* |  |
| Forward | AAC TGC TTC CCC CTC TGT TCT |
| Reverse | CTG CTC TGT GAG GCT GTT CAA |
| *GATA-3* |  |
| Forward | GGC ATC TGT CTT GTC CCT AT |
| Reverse | CAC GCT GGT AGC TCA TAC AC |
