# Supplementary material for: SERPINB10 contributes to asthma by inhibiting the apoptosis of allergenic Th2 cells
Source: Respir Res. 2021 Jun 14;22:178. doi: 10.1186/s12931-021-01757-1 (PMC8201873; doi:10.1186/s12931-021-01757-1)
Supplement: Supplementary file 2 — Additional file 2. The protocol of RT-qPCR and Western blotting. [file 12931_2021_1757_MOESM2_ESM.docx]

**Supplementary Materials and Methods**

**RNA isolation and real-time reverse transcription-quantitative polymerase chain reaction (RT-qPCR)**

Total RNA from lung tissues was isolated using TRIzol^®^ Reagent (TaKaRa Biotechnology, Shiga, Japan). Total RNA from polarized T cells was isolated using the RNeasy^®^ Micro Kit (Qiagen, Stanford, VA, USA). Total RNA was reverse-transcribed using the PrimeScript^®^ RT Reagent Kit (TaKaRa Biotechnology) according to manufacturer instructions. The transcription level of each gene was determined using SYBR^®^ Green Master Mix (Yeasen Biotech, Shanghai, China) and an ABI Prism 7500 PCR system (Applied Biosystems, Foster City, CA, USA). The primer sequences of all genes for PCR are listed in Supplementary Table S1. Fold differences were determined by the 2^–ΔΔCT^ method.

**Western blotting**

SERPINB10 protein in mouse lungs was measured by western blotting using polyclonal antibody for SERPINB10 (1:2000 dilution; Affinity Biosciences, Cincinnati, OH, USA). Antibody was detected using horseradish peroxidase-conjugated goat anti-rabbit immunoglobulin (Ig)G (1:4000 dilution; Aspen Chemicals, Bedford, MA, USA) followed by electrochemiluminescence western blotting detection reagent (Beyotime Biotech, Beijing, China). Protein expression of SERPINB10 was indexed to that of glyceraldehyde 3-phosphate dehydrogenase（GAPDH）.
